# The Impact of the COVID-19 Virus Pandemic on the Incidence of First Psychotic Spectrum Disorders

**DOI:** 10.3390/ijerph19073781

**Published:** 2022-03-22

**Authors:** Kacper Łoś, Joanna Kulikowska, Napoleon Waszkiewicz

**Affiliations:** 1 Department of Psychiatry, Medical University of Bialystok, 15-089 Bialystok, Poland; napwas@wp.pl; 2 Department of Neurology, Medical University of Bialystok, 15-089 Bialystok, Poland; joannaakulikowska@gmail.com

**Keywords:** COVID-19, SARS-CoV-2, psychotic spectrum disorders, health promotion, schizophrenia, mental disorders

## Abstract

The effects of COVID-19 on the human body are not yet understood enough. Medical history provides information on cases of psychiatric symptoms during viral infections in the 20th century, such as the influenza pandemic. Currently, it is observed that there is an increasing number of new psychiatric disorders in previously healthy individuals. In addition, because of the decreased amount of reporting to health care providers, including psychiatrists, many physicians suggest that the number of neuropsychiatric disorders may be underestimated. In this paper, we review available studies on the occurrence of first-time psychotic spectrum disorder (PSD) in individuals related to SARS-CoV-2 infection. The reviewed studies suggest that first-time psychotic disorder in COVID-19 patients is statistically significantly more frequent compared to influenza, as well as to other respiratory infections. The emergence of new PSDs is explained by direct neurotropism of the virus on the one hand and by immunological mechanisms on the other. The main conclusions of this review should be treated with caution, and future research on this topic is needed. The authors recognize the particular need to develop standardized laboratory panels that include inflammatory markers (IL-6, TNF-α), cerebrospinal fluid (CSF) testing, and SARS-CoV-2 antibody assays to entirely understand the etiology of neuropsychiatric complications of SARS-CoV-2 infections and the pandemic itself. In addition, public health efforts are required to promote mental health, especially during COVID-19.

## 1. Background

### 1.1. General Information

SARS-CoV-2 virus, a member of the beta-coronaviruses, is responsible for the COVID-19 pandemic, which was declared by WHO in March 2020 [1]. To date, more than 343 million people worldwide have been infected, of which 5.5 million have died. According to current statistics, 98% of infections are mild, while only 2% are severe [2]. The most common symptoms include fever, cough and shortness of breath [1]. Important is the course of the infection, which can be divided into several stages [3]. A particular key stage is period III, called immune storm, in which a very large amount of proinflammatory cytokines such as IL-1, IL-6 and TNF-α are released [4]. These mediators can contribute to damaging various organs, mainly the lungs [5]. Interestingly, since the beginning of the pandemic, extrapulmonary symptoms have also been observed, including features of nervous system damage and psychiatric symptoms [6]. There is direct evidence for the initiation of neurodegenerative processes in the central nervous system (CNS) as a result of SARS-CoV-2 infection, which may explain the occurrence of both neurological and psychiatric diseases [7]. However, the route of coronavirus entry into the CNS has not been fully clarified. On the one hand, direct invasion of the CNS by nerve endings such as the olfactory bulb, is speculated, and on the other hand, indirect CNS damage by immunological mechanisms [8,9].

### 1.2. Coronaviruses Impact

For many years, a group of coronaviruses has been suspected of causing psychosis, which was confirmed by the study of E. Severance et al., comparing seroprevalence of IgG antibodies to human coronaviruses 229E, HKU1, NL63 and OC43 in patients with psychotic symptoms and in the control group without psychotic symptoms. The study group showed statistically significantly higher seroprevalence of antibodies against the four studied coronaviruses, which is another indication that viral infections are a risk factor for the development of psychotic disorders [10]. Due to the enormous rate of spread of the pandemic, long-term, multicenter, case–control studies are needed, which include individuals who have undergone SARS-CoV-2 infection. To date, only a small number of studies have been published, with a short follow-up period after hospitalization [11].

### 1.3. COVID-19 Impact

An extremely interesting report is provided by a 6-month cohort study conducted on a population of 1733 patients, including patients discharged from the hospital who experienced COVID-19 infection. The main complaints observed were fatigue and muscle weakness in 63% of patients after discharge from hospital. The second most common complaint reported was difficulty in sleeping, occurring in 26% of subjects, while symptoms of anxiety and depression were observed in 23% of patients [11]. This may indicate a projected rapid increase in the prevalence of psychiatric disorders in the general population after this pandemic [12]. This is supported by the study of Taquet et al., which provides evidence for a high prevalence of neurological and psychiatric disorders within 6 months after COVID-19 [13]. Worth noting are the results of a clinical study conducted by Seung Won Lee et al. on a population of South Koreans, indicating that individuals with a mental health diagnosis had a slightly increased risk of severe COVID-19 compared with individuals without an associated mental disorder. In contrast, a diagnosis of mental illness did not increase the probability of being positive for SARS-CoV-2 [14]. This can be explained by the frequent social withdrawal resulting from negative symptoms among those with mental illness. Interestingly, there is emerging information regarding a positive correlation between exposure to the COVID-19 epidemic and the onset of new psychosis spectrum disorders (PSD) in previously mentally healthy individuals [15]. This develops questions about the role of environmental stressors in the induction of new PSD [16].

### 1.4. Aim

It is worth noting that in the early 20th century there was an increase in the incidences of psychosis. This is probably related to the influenza pandemic, during which the possibility of neuropsychiatric symptoms, secondary to viral infection, were noted [17]. Since then, new infectious theories of psychosis have emerged from time to time. The identification of such infectious factors that are involved in the etiopathogenesis of schizophrenia may result in new methods of preventing and treating the disease [18]. Furthermore, elevated viral antibody titers have been reported among schizophrenic patient populations. Hence, the question arises of if the current pandemic and associated exposure to coronavirus may cause neuronal inflammation in the central nervous system that initiates newly emerging psychotic symptoms [19]. The purpose of this review is to provide information regarding the impact of the COVID-19 pandemic on the occurrence of first psychotic spectrum disorders.

## 2. Materials and Methods

We reviewed the literature on the occurrence of first-time psychotic symptoms in previously healthy individuals. We searched the available literature in the databases PubMed, Scopus, and Google Scholar using keywords COVID-19, SARS-CoV-2, clinical features, consequences of COVID-19, psychosis spectrum disorders, mental illness, impact of COVID-19, hypothesis of schizophrenia, psychosis risk, first episode psychosis and various combinations of these keywords. For the review we included 38 papers, in English, that were published in peer-reviewed journals. These include 5 cohort studies and 13 case reports. We covered all studies that appeared from 2020 to the publication of this review. Additionally, we included relevant older articles that provide a background to the problem. We excluded from the review individuals who had a prior diagnosis of mental illness, or individuals who had a subsequent psychotic episode. We also excluded children and patients with organic damage of the central nervous system. In addition, we did not include individuals with disorders of consciousness. We included only patients with a first psychotic episode who had not previously received psychiatric treatment. The most important collected articles are summarized in Table 1.

## 3. Results

Recent neurobiological evidence and epidemiological data on previous pandemics link the occurrence of psychosis with a viral infection. This raises the question of whether there is a risk of developing first-time psychotic disorders during COVID-19 [30]. For example, during the 2002–2003 SARS-CoV-1 epidemic, complications associated with psychiatric disorders were observed. During the current pandemic, most attention has been focused on systemic complications, but more studies are providing data on potential neurotropic effects [31]. Such information is particularly important to be able to identify early and intervene effectively in the increasingly visible social and health impacts of the COVID-19 pandemic [32].

### 3.1. Cohort Studies

Rentero et al. report a large number of patients with COVID-19 who developed first psychotic symptoms. These patients had no prior psychiatric illness, and the symptoms were characterized by structured delusional beliefs. Authors note that this may be similar to the first-time psychotic episodes seen in schizophrenia. In addition, it was noted that among patients with delirium presence, psychotic symptoms continued to persist despite resolution of the disturbance of consciousness. Of great clinical importance is whether the psychotic episodes induced by SARS-CoV-2 infection are primary, stress-related in genetically predisposed individuals, or secondary, related to treatment or metabolic abnormalities occurring during COVID-19 [33]. A large population-based cohort study, retrospectively reviewing more than 200,000 patients, highlighted a significantly increased risk of psychotic disorders in the 6-month follow-up period of patients after COVID-19. This confirms previous authors’ suggestions of an increased risk of new psychotic disorders during COVID-19. Psychotic disorders were observed in 1.4% of patients, and the first psychotic episode by 0.42%. The incidence of first psychotic disorder in the course of COVID-19 is statistically significantly higher compared to both influenza and other respiratory infections (*p*-value < 0.0001) [13]. A subsequent observational study conducted in the United Kingdom included 153 patients with COVID-19. Of the reported population, 23 patients met the criteria for a psychiatric diagnosis, and in addition, 43% of them (10 patients) had a neuropsychiatric disorder defined as a first PSD. Interestingly, statistically, the primary psychiatric diagnoses were made for younger patients [20]. A retrospective and observational study conducted at Hospital 12 de Octubre (Spain), including all hospital patients during the March/April 2020 coronavirus wave, identified 16 COVID-19 patients with new psychotic symptoms. Those with a history of psychiatric disorders and those who met the criteria for the presence of delirium were excluded. The remaining 10 patients exhibited structured delusions (50%), auditory hallucinations (40%), and visual hallucinations (10%). Patients’ laboratory studies revealed elevated inflammatory markers CRP and ferritin. The authors of this cohort study suggest a psychotic episode, caused by CNS invasion with coronavirus, or a systemic hyper-inflammatory reaction as the cause of PSD [34].

### 3.2. Case Reports

In addition to large cohort studies, the case reports found in the literature are extremely valuable. One of the first documented cases of PSD with COVID-19 symptoms was a 36-year-old, previously healthy woman. The patient presented with a rapid change in behavior characterized by persecutory delusions. In the absence of alternative etiologies for the development of PSD, direct SARS-CoV-2 infection was considered to be the cause. Neither computed tomography (CT), magnetic resonance imaging (MRI) nor cerebrospinal fluid (CSF) examination revealed pathological changes. The hypothesis of delirium was also rejected in the differential diagnosis. The authors of the clinical case suggest that the diagnosis of COVID-19 and associated stress or viral psychosis may have been inducers of psychosis [19]. Another case reported in the medical literature is a 55-year-old woman, in whom initial psychotic symptoms were observed after SARS-CoV-2 infection. Observed complex delusions, visual and auditory hallucinations, and Capgras Syndrome were probably the result of delirium. However, despite the resolution of qualitative disturbances of consciousness, the psychotic symptoms did not disappear. Electroencephalography (EEG), MRI, CSF and autoimmune panel did not reveal any abnormalities. Only increased levels of tumor necrosis factor (TNF)-α and increased proinflammatory factors c reactive protein (CRP) and ferritin were noted. The authors of this case propose an inflammatory etiology of the observed psychosis or encephalitis caused by specific antibodies in response to viral infection. The patient had never had symptoms that might suggest a tendency toward psychiatric disorders prior to admission [21]. It is possible that PSD symptoms may have been provoked by psychological stress; however, the presence of elevated TNF-α may be associated with both primary and secondary psychotic episodes [35]. On the other hand, elevated levels of IL-6, which is characteristic of a primary psychotic episode, were not observed in this case [36]. Another case is a 43-year-old patient with no previous medical history but with an episodes of occasional cocaine use. The patient developed a sudden change in character after hospitalization for COVID-19. The authors must mention the case of a 39-year-old man who developed his first psychotic episode during hospitalization for COVID-19. In the absence of other risk factors, it is hypothesized that it was triggered by a cytokine storm associated with significantly elevated IL-6 levels (more than 10-fold) [22].

Aggressive behavior and vulgarity became noticeable. In addition, police intervention was required during hospital admission. The imaging examinations of the head (computed tomography, magnetic resonance imaging) and urine toxicology performed did not reveal any abnormalities. Apart from mild leukocytosis, no other inflammatory exponents were observed. The patient was discharged from the ward with a diagnosis of a substance/drug-induced manic episode with psychotic symptoms. This effect could have been caused by the patient’s intake of glucocorticosteroids [37] or the use of hydroxychloroquine [38] to treat COVID-19. On the other hand, a history of cocaine use despite a negative urine drug screen, is relevant in such a case [23]. An interesting case is a 30-year-old man who developed paranoid delusions and auditory hallucinations a week after his COVID-19 diagnosis. Interestingly, the patient had only mild symptoms of coronavirus infection and was not hospitalized. The psychiatrist examining the patient concluded that the beliefs presented by the patient met the criteria for delusions. Due to the sudden onset of new PSD, CT scans were performed and showed no abnormalities. Morphology revealed only mild leukocytosis. Due to the severe stress associated with the COVID-19 diagnosis, the patient was diagnosed with brief reactive psychosis. Both delirium and psychotic disorders secondary to somatic illness were excluded. The case of this patient suggests that PSD may also be associated with mild COVID-19 symptoms [27]. The author presented the case of a male patient in whom PSD was observed in the form of delusions and hallucinations. The patient, not previously treated psychiatrically, tested positive for SARS-CoV-2. Differential diagnosis excluded delirium and neurological etiology [24].

It has been also reported that an acute psychotic episode can occur not only in the acute phase of infection, but also in the recovery phase after COVID-19 infection. M. Chacko et al. presented the case of a 52-year-old man, with no previous psychiatric disorder, who was admitted to a psychiatric emergency room with mutism lasting for a week. It was known from the family that the man had manifested verbal delusions over the past few days. A CT scan of the head was performed, which was normal; elevated D-dimers were noted. A chest CT excluded pulmonary embolism but showed inflammatory changes in the lungs. PCR for COVID-19 was negative. After several days, the patient presented again to the psychiatric emergency room after attempting to cut his throat with a knife. He was admitted to the Psychiatric Unit, where he tested positive for IgG antibodies to COVID-19, indicating a recent infection [25].

Apart from the association of SARS-CoV-2 viral infection with the occurrence of first-time psychotic disorders, an important issue is the influence of social isolation itself on their possible induction. There were described three clinical cases of patients in whom an acute psychotic episode was preceded by social isolation, caused by the pandemic and excessive fear of coronavirus infection. All three patients were tested negative for SARS-CoV-2 [28]. The available literature also includes descriptions of three cases of people who were in quarantine, during which psychotic symptoms developed, such as delusions of lack of freedom, delusions of loss, ruin, guilt and destructive fury associated with visual hallucinations [29]. Similar experiences were reported by M.J. Valdés-Florido et al. in a series of four cases of patients hospitalized in a psychiatric hospital with reactive psychosis, which appeared during the first two weeks of compulsory national quarantine in Spain. The authors point out the increasing number of newly diagnosed psychoses associated with COVID-19 in previously sane people. What is extremely alarming, is that the authors of the above-mentioned study draw attention to the fact that this type of psychosis has a high risk of suicide and recurrence of psychotic disorders [26].

This literature review did not include individuals with a history of psychiatric illness, but an exacerbation of psychiatric disorders in the setting of coronavirus infection has been reported in the literature and therefore is worth noting.

## 4. Discussion

The literature cited in the results section highlights the occurrence of first-episode psychotic disorders induced by COVID-19 and emphasizes the need for further research. Worth mentioning here is Menninger’s research including 100 patients in whom neuropsychiatric complications were associated with influenza infection [39,40]. This shows that psychotic complications after viral infection have been hypothesized for a long time.

During the SARS pandemic in 2005, a relationship between coronavirus infection and the possibility of a psychotic episode was noticed. In a survey (*n* = 308) conducted by B. Sheng et al., risk factors for the occurrence of psychiatric disorders were identified, which turned out to be severe disease, high doses of glucocorticosteroids, and interestingly, being a health-care worker [41]. So far, no similar studies have been carried out in COVID-19 patients, but the current experience may indicate similar risk factors.

Current literature also contains case reports of new psychotic disorders being induced by the effects of the pandemic rather than directly by the coronavirus. In addition, noteworthy are reports of a high risk of suicide attempts in reactive psychosis manifesting during social isolation or quarantine.

From the cited literature we learn that further research on this issue is needed, especially studies that would include longer follow-up of patients, their treatment and its effects. Taquet et al. show that within 6 months after SARS-CoV-2 infection in 0.42% of individuals, the first psychotic episodes appeared and were statistically more frequent than in influenza and other respiratory infections [13]. Among the COVID-19 patients studied, structured delusions appeared to be the most common psychiatric symptoms [19]. In searching for an etiologic hypothesis for new PSDs, the authors noted two main directions. The first is direct neurotropism of the virus into the CNS. The second is an immunological hypothesis in which autoimmune encephalitis and CNS damage due to systemic inflammatory reaction can be distinguished [8]. Unfortunately, there are no established standards or panels of tests used in the care of COVID-19 patients with the first-time PSD. It was noted that only a small number of cases have had CSF testing performed. Although the examination of the cerebrospinal fluid, including the autoimmune panel, remained without abnormalities in the described cases, the appearance of as-yet-unidentified autoantibodies that may be induced by the presence of the new coronavirus cannot be excluded [42]. In addition, not all patients had Il-6, or TNF-α levels tested, which makes it very difficult to draw conclusions about the etiology of new PSDs.

It should not be forgotten that psychosis can also be induced by the use of high doses of steroids, which are an integral part of COVID-19 treatment. A meta-analysis by J.P. Rogers et al., who examined the incidence of neuropsychiatric disorders in COVID-19, found that steroid-induced psychosis occurred in 0.7% of the study population during the acute phase of SARS-CoV-2 infection [43].

The authors of this review postulate to search for a panel of tests that could help to explain the etiology of new psychotic disorders in COVID-19 patients. Noteworthy tests are inflammatory markers (including Il-6, TNF-a), CSF examination (general examination, antibodies to SARS-CoV-2, PCR against SARS-CoV-2) and head imaging, often already used in cases of new PSD [42]. It is also worth noting a fact that may significantly affect the statistics. Numerous emerging studies, as well as the experience of the authors of this review, suggest that during the SARS-CoV-2 pandemic, patients avoided access to hospital health care [44]. The reduction in hospitalizations may not show the magnitude of the growing problem, which should mobilize researchers to study the issue of the first-time PSD more thoroughly. On the other hand, public health efforts on mental health promotion are needed, such that patients should not underestimate serious mental health problems. Early diagnosis and appropriate treatment are especially important in diseases such as schizophrenia. In addition, concerning the need for antipsychotic medications in COVID-19 patients, it is worth citing studies on medication safety. The authors state that special attention should be paid to drug interactions between psychotropic drugs and antiviral drugs. Zhang et al. states the relative safety of olanzapine and valproate when treatment with the above-mentioned group of drugs is required [45]. Moreover, the COVID-19 pandemic may not only have a deleterious effect on people who have not previously been mentally ill, but also adversely affect the environment of vulnerable people, especially those who have already developed prodromal symptoms, or patients already diagnosed with schizophrenia [16]. It is also important to differentiate paranoid psychotic disorder from affective psychotic disorder, that has mood-congruent features, and paranoia from paranoic psychotic disorder that has systematized features. Careful management involving an understanding of the mechanisms and trends of PSD is particularly important in the pandemic era and immediately thereafter [46]. Accurate tracking of subsequent symptoms over time will be the most important element in predicting the effects of SARS-CoV-2 infection on the CNS [32].

## 5. Conclusions

This article reviews the available research on the incidence of first psychotic disorders in individuals due to SARS-CoV-2 infection. The review of studies shows that this is a notable problem; moreover, first psychotic disorders in patients with COVID-19 are statistically significantly more common than in influenza and other respiratory infections. The main conclusions of this review should be treated with caution and future studies on this topic are needed. The authors recognize a particular need to develop standardized laboratory panels to fully understand the etiology of neuropsychiatric complications of SARS-CoV-2 infections and the pandemic itself.

## Figures and Tables

**Table 1:** The Impact of the SARS-CoV-2 Virus on the Incidence of First Psychotic Spectrum Disorders.

Cohort Studies	Case Reports	Excluded
[13]	[20,21,22,23,24,25,26]	-prior diagnosis of mental illness -subsequent psychotic episode -children -patients with organic damage of the central nervous system -individuals with disorders of consciousness
The Impact of COVID-19 Quarantine and Fear		
[27,28,29]		

## Data Availability

Not applicable.
